# Development and characterization of an on-chip glioma outgrowth model

**DOI:** 10.1007/s44164-025-00100-y

**Published:** 2025-12-08

**Authors:** Joshua P. A. Daoud, Alexandra E. Collisson, Daniya Asaid, Ester Clarisse do Couto Lopes, Dimitrios Paraskevopoulos, Christopher A. R. Chapman

**Affiliations:** 1 School of Engineering and Materials Science, Queen Mary University of London, London, UK; 2 Blizard Institute, Queen Mary University of London, London, UK; 3 Department of Neurosurgery, The Royal London Hospital, Barts Health NHS Trust, London, UK

**Keywords:** Glioma, Microfluidics, Lab-on-a-chip, Co-culture, Cancer models, Bioengineering

## Abstract

Tumour recurrence in high-grade gliomas, such as glioblastoma, is extremely common due to the challenges associated with complete surgical removal. Approximately 95% of glioblastoma tumours recur within 2 cm of the tumour resection margin; however, unfortunately, the current clinical measurement techniques do not accurately capture cell state during critical stages of recurrence. The development of in vitro models for investigating glioblastoma recurrence outgrowth can inform further understanding of the mechanistic underpinnings of this process at a cellular level. This work presents the development of an on-chip in vitro platform which can be used to assess glioblastoma interactions with healthy neural cells. Using a custom single-channel multi-well microfluidic system, spheroids of GFP transfected rat glioma cells were co-cultured together with rat astrocytes to investigate differences in glioblastoma outgrowth between co-culture and monoculture (i.e. glioma only, or astrocyte only). Quantitative image analysis demonstrated significantly increased outgrowth of glioma cells from spheroids in co-culture compared to monoculture. Additionally, comparison of upregulation of markers associated with glioma aggressiveness (CD44) and astrocyte reactivity (glial fibrillary acidic protein) showed significant differences in cellular expression between the cells in co-culture and monoculture configurations. These findings align with existing literature suggesting that astrocytes facilitate a supportive environment for glioblastoma cells to proliferate and invade healthy tissue by changing to a reactive phenotype. Overall, this work presents a promising on-chip in vitro model that has the potential to aid in the quantification of glioblastoma invasion through directional control of outgrowth and offers the potential for the addition of complexities to more accurately model the glioblastoma neural cell microenvironment.

**Supplementary Information:**

The online version contains supplementary material available at https://doi.org/10.1007/s44164-025-00100-y.

## Introduction

Glioblastoma multiforme, a high-grade glioma, is the most common intrinsic malignant brain tumour diagnosis in adults [1]. Patient outcomes are notoriously poor with a 5-year survival rate of less than 7% [2]. These tumours are characterized as being substantially invasive, with an extremely high recurrence rate, as total surgical resection is challenging. Due to the propensity for this cancer to recur, patient deterioration is inevitable, even after several repetitions of chemotherapeutic and radiotherapy treatments [3]. Current practice relies on outpatient magnetic resonance imaging (MRI) or computed tomography (CT) scans to detect local cancer recurrences after surgical resection and inform neurooncological treatment [4]. Unfortunately, the time in between these scans is often too long to identify small changes in the tumour margin and rapidly inform clinician action thus leading to worsened patient outcomes [5, 6]. The development of new in vitro models that recapitulate the neural microenvironment during glioblastoma recurrence have the potential to provide key mechanistic understanding and feedback on treatment efficacy [7].

Current knowledge of the cellular mechanisms underpinning glioblastoma recurrence is limited by challenges in recording from patients and incomplete recapitulation of the healthy neural environment in glioblastoma organoid models [8]. Recently, three-dimensional spheroid models of gliomas have seen an increase in popularity in their use to simulate tumour behaviour [9]. These models have been shown to retain near-identical genetic expression to the original tumour [10] which enables researchers to investigate cellular processes as they would naturally occur in the brain [11, 12]. However, most of the developed spheroid models are focused on enabling more realistic testing of chemotherapeutic efficacy for drug discovery. Only a limited amount of research has been focused on developing models that fully recapitulate the neural environment in which glioblastoma occurs. Without these interactions it is challenging to accurately investigate the complex interactions between glioma cells and healthy neural cells like astrocytes, microglia, and neurons [12]. There is clear evidence that the interactions between glioma and healthy neural tissue is a critically important component to the efficacious treatment of gliomas, therefore it is essential to examine their hallmarks using in vitro models to simulate in vivo growth conditions [13] The development of a platform to investigate infiltration and recurrence of glioblastoma in a co-cultured environment consisting of healthy neural cells alongside glioma cells has the potential to facilitate further understanding of the mechanisms behind tumour recurrence [14].

Microfluidic platforms hold promise for providing an attractive medium between the complexities of animal models and limitations of traditional culture systems through specifically engineering the cell culture environment [15]. The rationale of this work was to produce a customizable in vitro model to enable the co-culture of a glioma spheroid with healthy neural tissue and constrain spheroid growth to aid in studying glioma margin outgrowth. From this model, the primary aim was to analyse tumour outgrowth, cell density, and key protein expression between healthy and cancerous cells during different stages of outgrowth. This work highlights that this in vitro model system provides an advantageous platform over traditional (i.e. non-constrained) cell models by providing an easily adaptable system to measure glioma outgrowth.

## Materials and methods

### Micropatterning

**Fig. 1:**
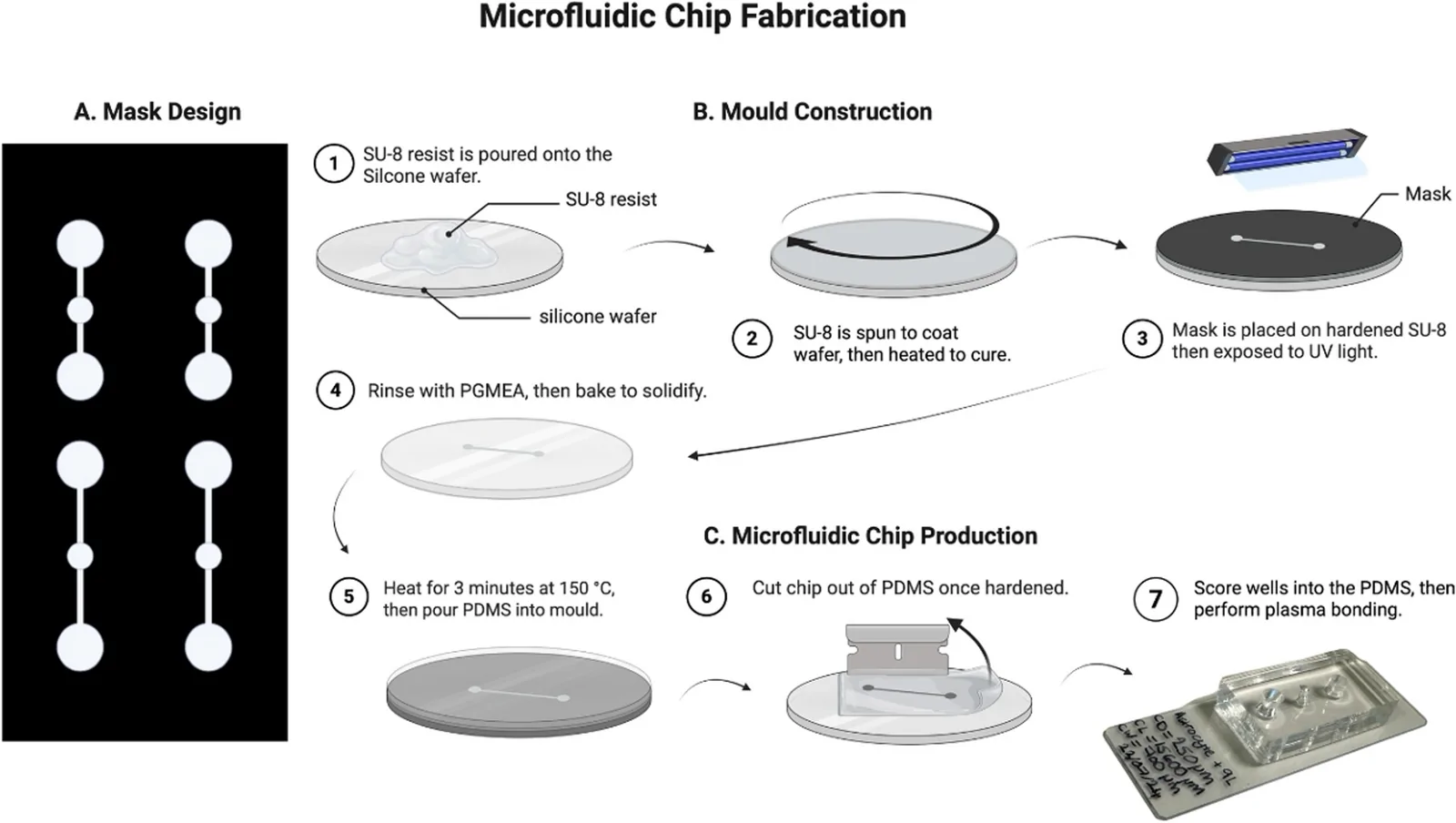
(**A**) Photomask design showing 4 microfluidic channels. (**B**) Mould construction stages showing how microfluidic channel moulds were fabricated using photolithography. (**C**) Stages of microfluidic chip production using the channel mould and Polydimethylsiloxane (PDMS) which was then plasma bonded onto a glass microscope slide

Photolithography masks for the microfluidic channels were designed using computer-aided design software AutoCAD (Fig. 1A) and purchased from Microlitho (UK). Channel moulds were created by spin coating SU-8 photoresist (Kayaku) at 500 rpm for 5 s, then 1000 rpm for 30 s onto blank silicon wafers and exposing for 8 s at 100% power (360 mW/cm^2^) with a 365 nm UV source (KLOÉ). After developing, masks were rinsed with Propylene Glycol Monomethyl Ether Acetate (PGMEA – Sigma-Aldrich) and baked at 150 °C for 3 min (Fig. 1B). After mould creation, microfluidic channels were fabricated from cast Sylgard-184 Silicone Elastomer (Farnell UK) at 80 °C for 1.5 h (Fig. 1C). Channel dimensions were then analysed using an optical profilometer (Omniscan), then visualised in Profilm3D.

### Cell culture

#### Cell preparation

The 9L/3cmv-GFP glioma cell line, were previously transfected with green fluorescent protein (GFP) and donated from the laboratory of Prof. Rylie Green at Imperial College London. The CTX-TNA2 astrocyte cell line were procured from UK Health Security Agency. Both cell lines were established and cultured in Dulbecco’s Modified Eagle Medium (DMEM), with 10% fetal bovine serum (FBS) and 1% penicillin/streptomycin (Pen/Strep) all purchased from ThermoFisher Scientific. Cells were incubated at 37 °C and 5% CO_2_, with passaging performed every 4–5 days.

#### Spheroid preparation

5 days before co-culture experiments, 9L/3cmv-GFP cells were grown into spheroids using a Corning 96-well spheroid-forming non-adherent microplate (Sigma-Aldrich) and DMEM media. Media was replenished on day 3 to optimise viability and minimise media loss through evaporation.

#### Microfluidic preparation for cell culture

First, microfluidic channels were sterilized using autoclave sterilization at 121 °C and flushed with sterile phosphate buffer solution (D-PBS). Microfluidic channels were coated using a solution which consisted of 25 µg/mL Poly-D-Lysine and 10 µg/mL Laminin, which provided improved adhesion of astrocytes. No further growth factors were added to prevent any interference with cell-cell interactions [16]. Full coating protocol is provided in Supplementary Information. Figure S1 shows additional features of the microfluidic chip design, including rings which were cut from a flat disc of cured PDMS and adhered onto the upper surface of the chip using KORASILON silicone paste (Sigma-Aldrich), as this created a reservoir of DMEM media.

#### Outgrowth experiments

##### Co-culture

Astrocyte cell suspension was added into each microfluidic chip at a seeding density of 50,000 cells/mL. Previous optimisation experiments demonstrated that a higher seeding density could not be sustained for 14 days due to becoming overly confluent. This was achieved by adding 400 µL of astrocyte cell suspension into both ends of the microfluidic channel, which gave a total added volume of 800 µL. This ensured an even distribution of cells throughout the channel.

After the addition of astrocytes, one glioma spheroid was added into the centre well of the microfluidic channel. Co-culture samples were incubated at 37 °C and 5% CO_2_ and allowed to grow without interruption for 48 h to allow cells to adhere. After the initial 48-hour period, the first media change was performed using fresh media, then after 96 h, a second media change was performed. After the 96-hour period, subsequent media changes were performed daily to prevent the buildup of cell debris. Co-culture experiments were grown until two timepoints: 7 days and 14 days. Due to the high rate of evaporation in these low volume systems, media changes were optimised to reduce build-up of cell debris and maximise cell survival over the length of the cell culture period (Figure S2). There was slight variability in the placement of the glioma spheroid in the well after transfer (Figure S7).

##### Monoculture

Monoculture experiments consisted of glioma spheroids grown in isolation and CTX-TNA2 astrocyte cells grown in isolation inside of the microfluidic platform. These samples were also grown for 7 and 14 days and received the same media replenishment schedule as co-culture samples.

### Cell staining and imaging

Three staining protocols were carried out, one for outgrowth experiments and another for CD44/GFAP expression experiments, and a third for dead cell staining the spheroids. Full staining protocols are provided in the Supplementary Information.

In all experiments, 9L/3cmv-GFP and CTX-TNA2 cells were washed three times with phosphate-buffered saline (D-PBS), before being fixed inside of microfluidic chips using 4% paraformaldehyde (ThermoFisher Scientific) and subsequently permeabilised with 0.1% Triton-X solution (ThermoFisher Scientific).

#### Dead cell staining

Spheroids were stained using Live-or-Dye NucFix™ Red Staining Kit (Biotium), which was used to localise dead cells within the spheroid.

#### Outgrowth staining

For the outgrowth experiments, the glioma cell line already expressed GFP, so no green stain was used. Texas-Red Phalloidin (ThermoFisher Scientific) and 4’,6-diamidino-2-phenylindole (DAPI) (ThermoFisher Scientific) were used to selectively stain F-actin filaments and nuclear DNA in red and blue respectively.

#### Antibody staining

Cells were stained for CD44 using an anti-CD44 Recombinant Rabbit Monoclonal Antibody (Invitrogen) and GFAP using an anti-GFAP Mouse Monoclonal Antibody (GA5) (Invitrogen). Primary antibodies were visualised with secondary antibodies Cy™5 AffiniPure Goat-Anti-Rabbit IgG for CD44 (Jackson ImmunoResearch) and Cy™3 AffiniPure Goat-Anti-Mouse IgG for GFAP (Jackson ImmunoResearch).

### Analysis

Imaging was performed using fluorescent confocal microscopy with the Nikon CSU-W1 SoRa Spinning Disc Confocal microscope. Images were then viewed and processed using the Nikon Elements Viewer software. After processing, fluorescent images were analysed using ImageJ software.

Measurements of spheroid outgrowth distance were taken from fluorescent images of the co-culture and monoculture samples. Total outgrowth was calculated from fluorescent images in the ImageJ program by measuring the furthest detectable outgrowth of glioma cells from the 9L/3cmv-GFP spheroid (Figure S3). Measurements of cell density were also calculated for all samples (Figure S4).

Antibody staining images were also analysed using ImageJ to determine the relative intensity of antibodies in each sample. Relative intensities were calculated with ImageJ from individual cell types using counterstaining to delineate cell types (Figure S5).

### Statistics

GraphPad Prism software was used to carry out inferential statistical tests including unpaired and paired sample t-tests, and Mann-Whitney U tests if equal variances could not be assumed. Unless otherwise stated each experiment had a sample size of *n* = 5. Cell density data was obtained from an average cell counts per 0.2mm^2^ area, which was scaled up by a factor of 5 to produce values for number of cells/mm^2^. Antibody data measured relative colour intensity across whole cells which were repeated across all cells in each image and averaged to provide a mean intensity value. Power calculations suggest that for a type I error rate (α) of 0.05 and power (1 – β) of 80% a sample size of *n* = 5 enabled a minimum detectable effect (MDE) of ~ 1/3 of the arithmetic mean assuming a standard deviation of less than 1/3 of the mean, which is representative of the data in this study. This calculation supports the use of five independent in vitro model systems for each output in this study.

## Results

### Microfluidic platform dimensions

**Fig. 2:**
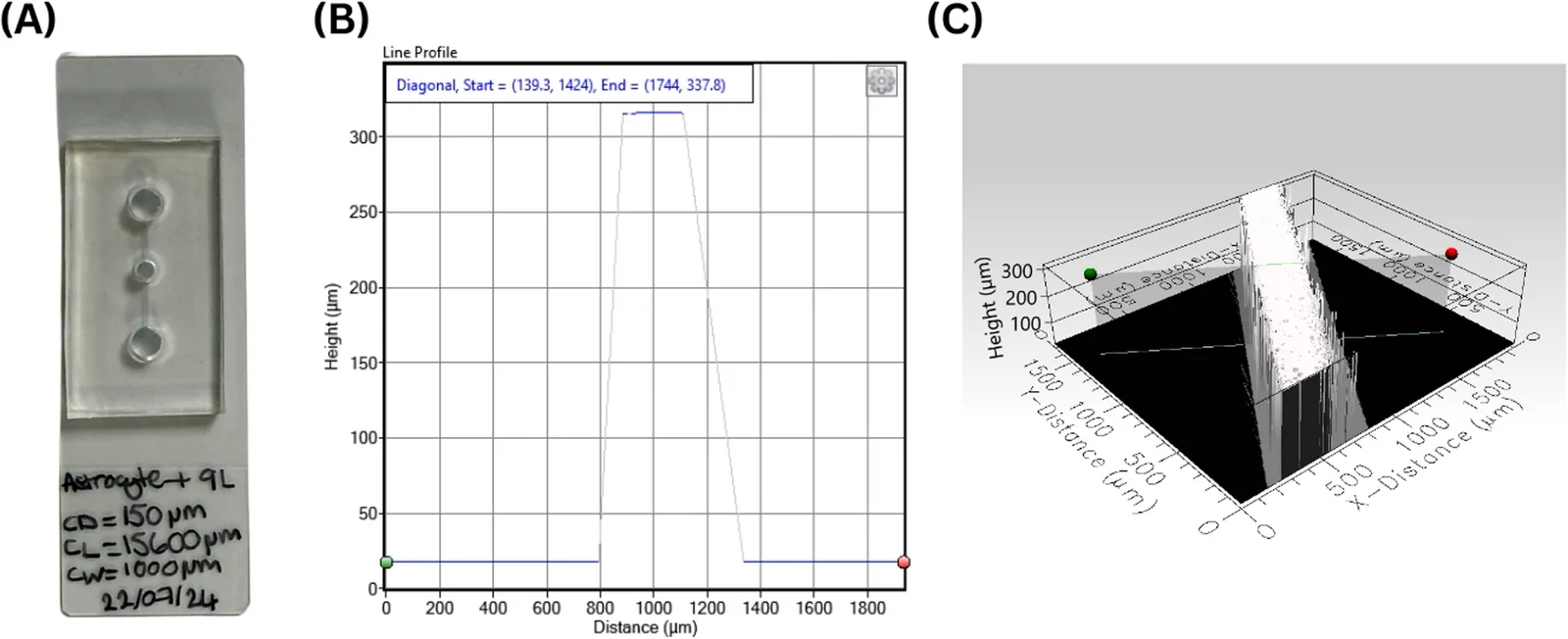
(**A**) Image of microfluidic chip, (**B**) the channel dimensions, showing a projection of channel height and width, and (**C**) profilometer measurements of microfluidic channel dimensions in Profilm3D

Media loss by evaporation is a common issue in microfluidic cell culture, which can lead to drastic differences in data as a result, particularly with the periphery of cell culture vessels [17]. To counter this issue and improve data reliability, channel dimensions of 15,600 μm (length) x 300 μm (depth) x 500 μm (width) were created to support larger media volumes (Fig. 2A). Final dimensions for the microfluidic channel (Fig. 2B and C) were confirmed by profilometer to be 15,600 μm (length) x 300 μm (depth) x 500 μm (width).

### Glioma spheroid optimisation

Spheroids were grown from the 9L/3cmv-GFP rat glioma cell line. Four different seeding densities were investigated for their overall growth patterns over days 1, 2, 5, and 7 in vitro. Overall spheroid diameter was tracked over time for each seeding density. Additionally, the formation of a necrotic core was targeted as a key timepoint in spheroid formation. This is often characterised by a collapsing of the spheroid diameter, and subsequent regrowth [18].

**Fig. 3:**
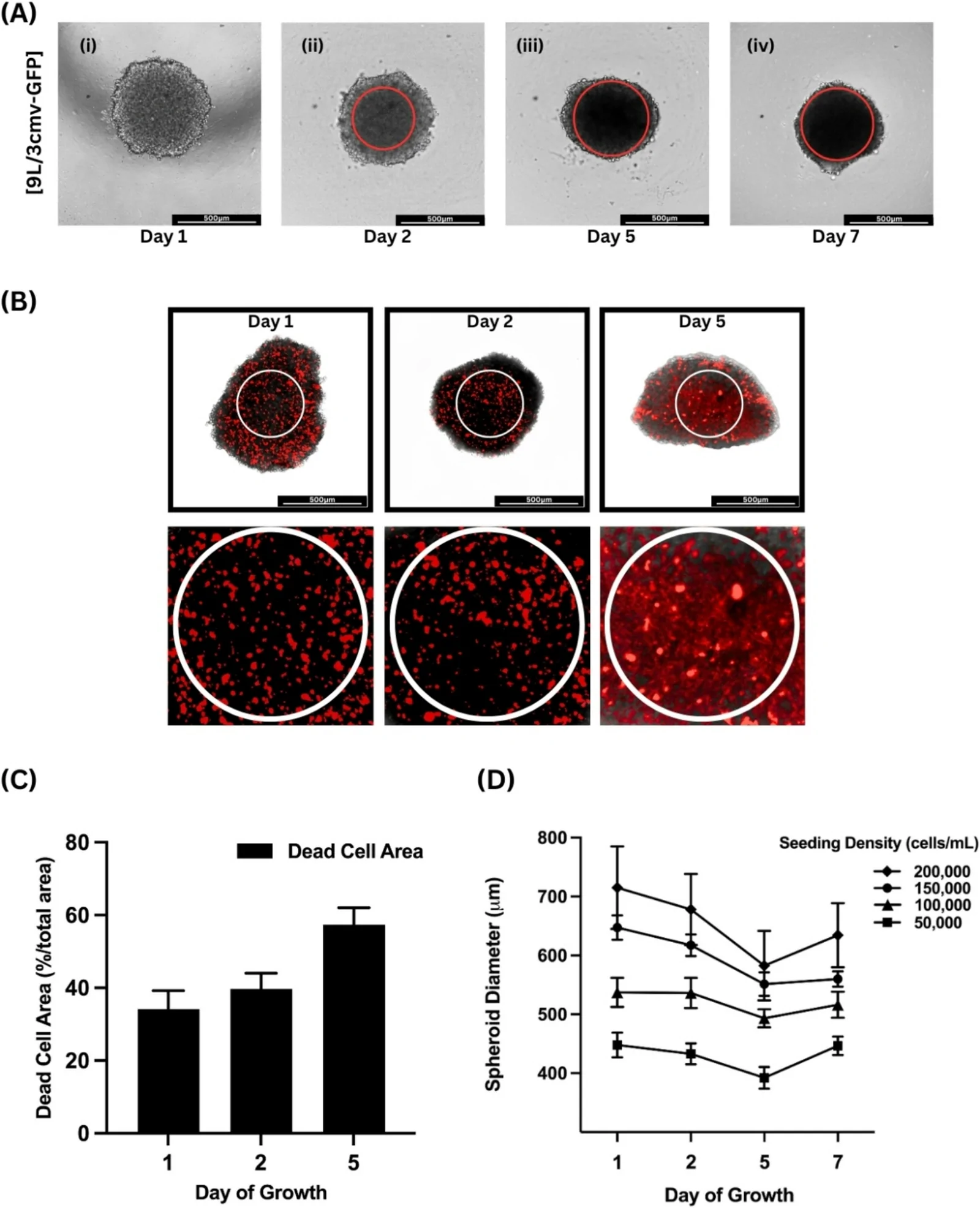
(**A**) Brightfield images taken at 10x magnification of 9L/3cmv-GFP spheroid growth at a seeding density of 150,000 cells/mL (i-iv). Red circle highlights the growing core structure. Scale bars represent 500 μm. (**B**) Localisation of dead cells (red) in 9L/3cmv-GFP spheroids. Images taken at 10x magnification are full stack images of the entire spheroid area. Scale bars represent 500 μm. (**C**) Calculations of dead cell area on days 1, 2, and 5 of growth in a 150,000 cells/mL density spheroid. Error bars represent standard deviation; *n* = 3. (**D**) Average spheroid diameters measured over time for each cell density. Error bars represent standard deviation; *n* = 4

Brightfield images of the spheroids (Fig. 3A) showed an increasingly dense and dark formation of cells in the spheroid centre (red circle), a feature described in existing literature as spheroids form a necrotic core [18, 19]. This increase in cell death at the spheroid core was confirmed by dead cell staining (Fig. 3B). Analysis of the dead cells in the spheroid showed a clear increase in dead cell coverage from day 1 to 5 (Fig. 3C). Additionally, a proportional relationship was seen between cell seeding density and spheroid diameter (Fig. 3D). Based on the microfluidic platform designed for this model (Fig. 2) the desired spheroid diameter was 500–600 µm to enable tight fit of the spheroid into the middle well (Figure S7). A decrease in spheroid diameter was observed for all seeding densities at day 5, followed by an increase. This is consistent with previously published data suggesting that initial tumour growth resulted in a loss of tumour cell viability, which is then closely followed by an increase in tumour diameter [20]. After necrotic core formation, glioma spheroids typically do not experience increased necrotic core diameters, and any increases in overall spheroid diameter after core formation result from peripheral tumour cell outgrowth [21]. A seeding density of 150,000 cells/mL was selected as the optimal target for co-culture experiments as this seeding density produced an appropriate spheroid size, with a forming necrotic core after 5 days and showed reduced variability compared to those grown with 200,000 cells/mL. The increased variability seen in the 200,000 cells/mL spheroids may have been caused by reaching a critical limit for the diffusion of nutrients into the spheroid leading to larger collapse in some of the formed spheroids.

### Glioma outgrowth in co-culture and monoculture

**Fig. 4:**
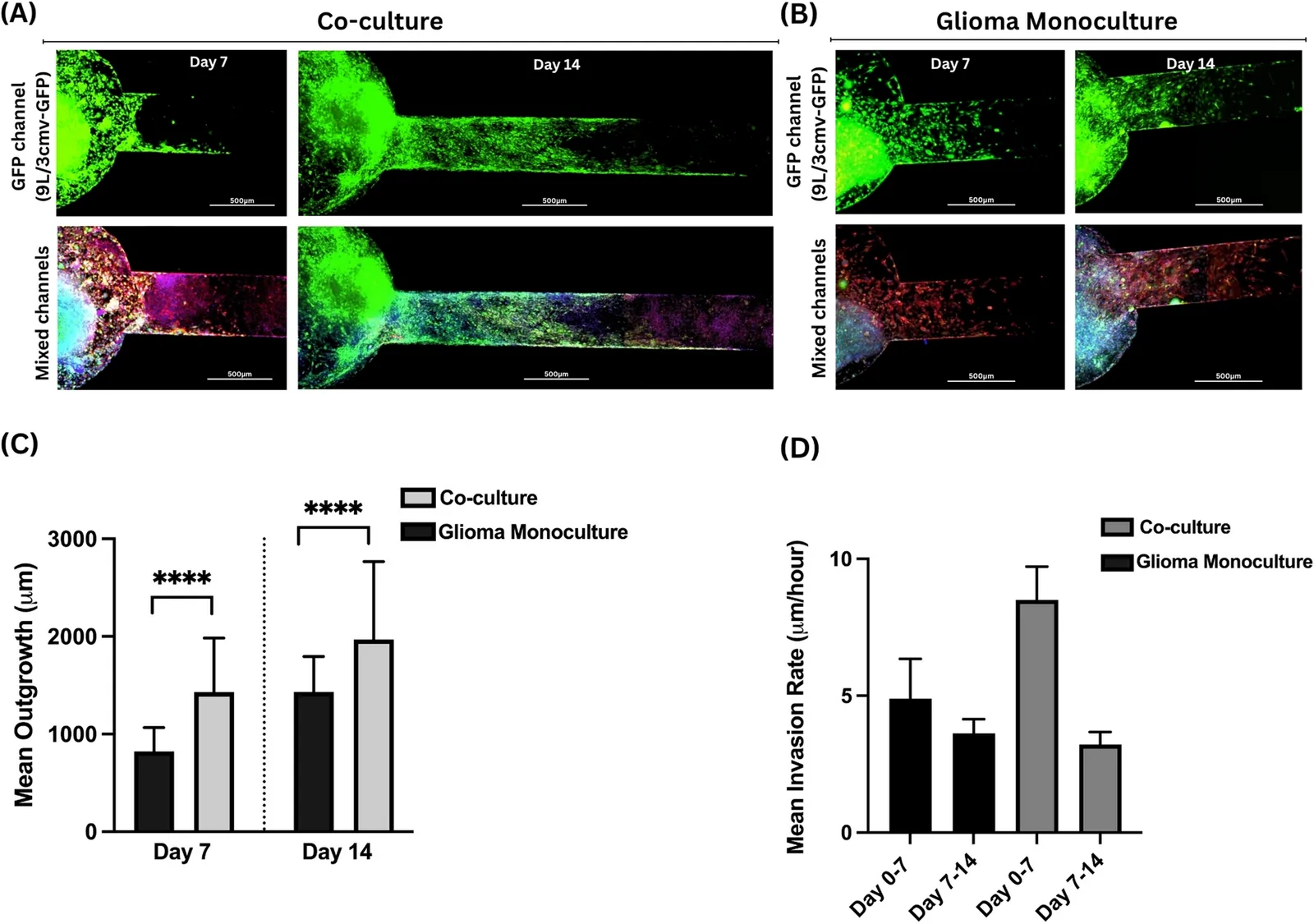
(**A**) Wide field images at 10x magnification of day 7 and 14 co-culture samples showing (top) GFP channel (green - glioma cells only), and (bottom) mixed channels including DAPI (dark blue - nucleus) and phalloidin (red – F-actin). Scale bars represent 500 μm. (**B**) Wide field images at 10x of day 7 and 14 glioma monoculture samples showing (top) GFP channel (green - glioma cells), and (bottom) mixed channels including DAPI (dark blue - nucleus), and phalloidin (red – F-actin). Scale bars represent 500 μm. (**C**) Mean glioma outgrowth (µm) for co-culture (grey) and glioma monoculture (black). Error bars indicate standard deviation; ****p < 0.0001, *n* = 5. (**D**) Mean rate of invasion of glioma cells in monoculture and co-culture for day 0–7 and day 7–14. Error bars indicate standard deviation; *n* = 5

Figure 4 highlights the differences in spheroid outgrowth distance between co-culture samples (containing glioma and astrocytes), and the glioma spheroid monoculture samples. In co-culture samples, the mean outgrowth was measured to be 1428 ± 556 $\:\mu\:$m on day 7, which increased to 1969 ± 796 $\:\mu\:$m by day 14 (Fig. 4A). In the glioma spheroid monocultures, the mean outgrowth was measured at 823 ± 243 $\:\mu\:$m on day 7 which increased to 1433 ± 342 $\:\mu\:$m by day 14 (Fig. 4B). Significant differences in outgrowth distance were observed between co-culture and glioma monoculture samples across both day 7 and day 14 timepoints (*p* < 0.0001) (Fig. 4C). Mean outgrowth data for co-culture samples showed a percentage difference of + 73.5% compared to glioma monocultures on day 7, and + 37.4% on day 14. From this data, an overall rate of outgrowth for each timepoint (i.e. day 0–7 and day 7–14) was calculated (Fig. 4D). The elevated rate of initial outgrowth in co-culture samples compared to controls is in line with glioma co-culture data which has been reported previously [22].

### Cell density

**Fig. 5:**
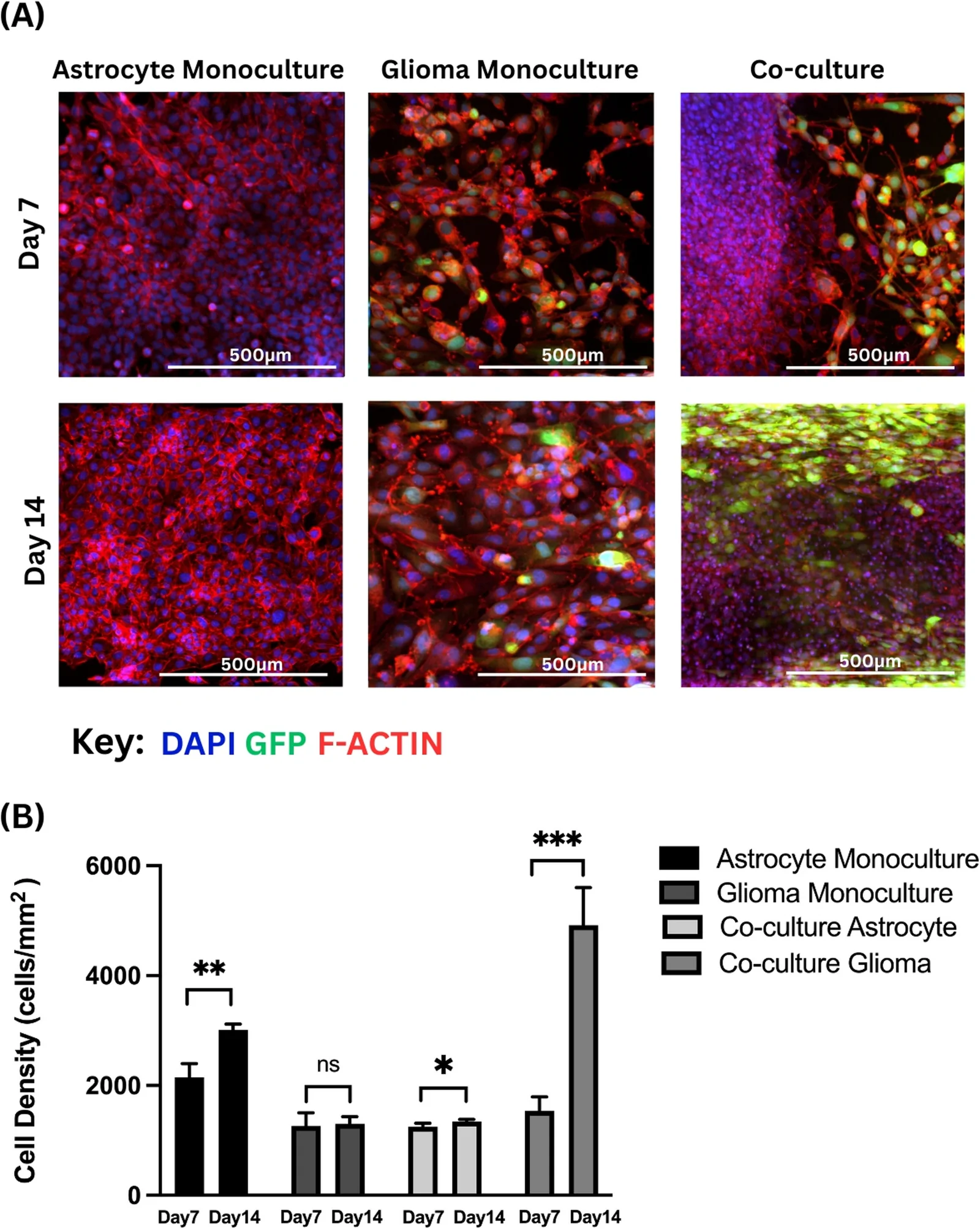
(**A**) Representative images at 20x magnification of astrocyte monoculture, glioma monoculture, and co-culture samples visualising cell nucleus (DAPI – blue), glioma cells (GFP – green), and f-actin (phalloidin – red). Scale bars represent 500 μm. (**B**) Calculated mean cell density (cells/mm^2^) for individual cell types (glioma and astrocytes) in both co-culture and monoculture; **p = 0.0033, *p = 0.0335, ***p = 0.0007, ns = non-significant, *n* = 5

Cell density was measured for both co-culture and monoculture samples (both glioma only and astrocyte only) along the length of the microchannel at days 7 and 14 (Fig. 5A). Significant changes in cell density were seen between glioma cells in co-culture and monoculture, as well as astrocytes in co-culture and monoculture (Fig. 5B). Glioma cells in co-culture showed a significant increase in cell density whereas monoculture glioma cells remained constant between day 7 and 14. The mean cell density was calculated in co-cultured glioma cells to be 1535 ± 258 cells/mm^2^ on day 7, which increased to 4918 ± 683 cells/mm^2^ by day 14 (*p* = 0.0007), whereas for glioma cells in monoculture the mean cell density was calculated to be 1261 ± 242 cells/mm^2^ on day 7 which non-significantly increased to 1300 ± 131 cells/mm^2^ by day 14 (*p* = 0.7784). Interestingly, the reverse phenomenon was seen between the co-culture and monoculture of astrocytes. The mean cell density for co-cultured astrocytes was 1245 ± 68 cells/mm^2^ on day 7, which increased minimally to 1340 ± 43 cells/mm^2^ by day 14 (*p* = 0.0335), whereas astrocytes in monoculture increased from a density of 2145 ± 254 cells/mm^2^ on day 7, to 3015 ± 101 cells/mm^2^ by day 14 (*p* = 0.0033), a much larger increase in density than what was seen in co-culture.

### CD44 and GFAP expression

**Fig. 6:**
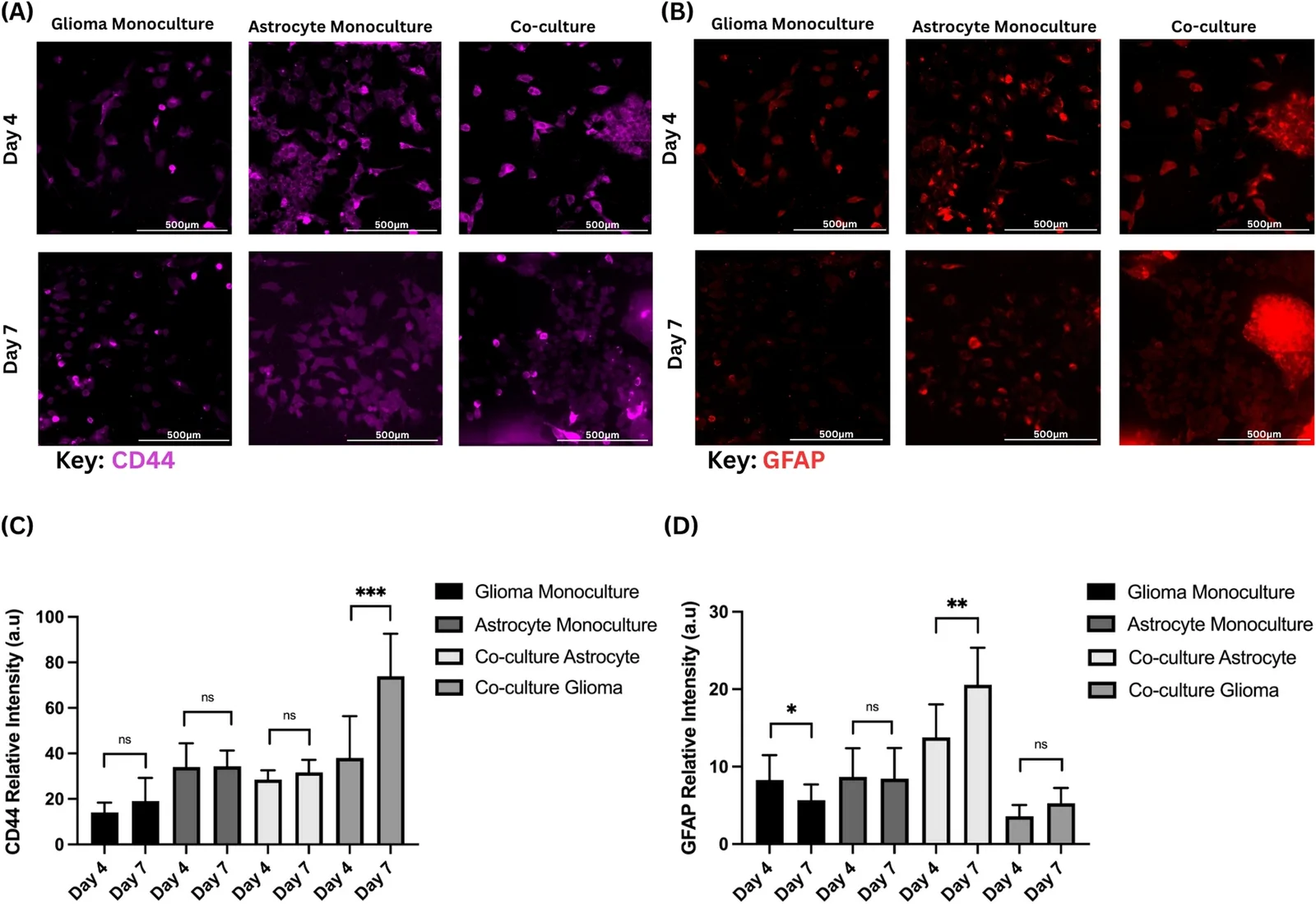
(**A**) Representative images taken at 20x magnification of astrocyte monoculture, glioma monoculture, and co-culture showing CD44 (purple). Scale bars represent 500 μm. (**B**) Representative images taken at 20x magnification of astrocyte monoculture, glioma monoculture, and co-culture showing GFAP (red). Scale bars represent 500 μm. (**C**) CD44 relative intensity per cell type in monoculture and co-culture. Error bars represent standard deviation; ***p = 0.0004, *n* = 5. (**D**) GFAP relative intensity per cell type in monoculture and co-culture. Error bars represent standard deviation; *p = 0.0459, **p = 0.0068, *n* = 5

Expression of key markers involved in glioblastoma outgrowth (CD44) (Fig. 6A) and astrocyte reactivity (GFAP) (Fig. 6B) were quantified across all co-culture and monoculture configurations (i.e. co-culture and glioma/astrocyte monocultures), at days 4 and 7.

A statistically significant increase in relative intensity of CD44 was only seen in glioma cells in co-culture with astrocytes (Fig. 6C) with an increase in relative intensity from 38 ± 18 (a.u) on day 4 to 74 ± 19 (a.u) by day 7 (*p* = 0.0004). Interestingly, the relative intensity of CD44 for the glioma monoculture samples were non-significant with a small increase in relative intensity from 14 ± 4 (a.u) on day 4 to 19 ± 10 (a.u) by day 7 (*p* = 0.1764). Astrocytes from the co-culture showed a non-significant increase in relative intensity from 28 ± 4 (a.u) on day 4 to 32 ± 6 (a.u) on day 7 (*p* = 0.1660), and the relative intensity of CD44 in astrocyte monoculture samples was also non-significant, at 34 ± 10 (a.u) on day 4 and 34 ± 7 on day 7 (*p* = 0.9317).

A statistically significant increase in relative intensity of GFAP was only seen in astrocytes in co-culture with glioma cells (Fig. 6D) with the relative intensity increasing from 14 ± 4 (a.u) on day 4 to 20 ± 5 (a.u) on day 7 (*p* = 0.0068), while glioma cells in the co-culture showed a slight non-significant increase in GFAP relative intensity from 4 ± 1 (a.u) on day 4 to 5 ± 2 (a.u) on day 7 (*p* = 0.7701). The relative intensities of GFAP in the astrocyte monoculture timepoints were non-significant, as the relative intensity remained at 8 ± 4 (a.u) for day 4 and 8 ± 4 (a.u) for day 7 (*p* = 0.8975). Surprisingly, there was a significant decrease in GFAP relative intensity for glioma cell in monoculture from 8 ± 3 (a.u) on day 4 to 5 ± 2 (a.u) on day 7 (*p* = 0.0459).

## Discussion

### Mechanistic insights of glioma outgrowth

The substantial outgrowth distance from glioma spheroid in the microfluidic channels when co-cultured with astrocytes is supported by existing research which suggests that astrocytes undergo malignant transformation into a more tumour-permissive phenotype, which may facilitate a supportive environment which promotes glioma invasion [23]. The results presented here indicated that after prolonged contact with astrocytes in a co-culture environment, glioma cells showed a relative increase in intensity of CD44, which suggests that increased contact with astrocytes in this model may have contributed to the invasive properties of the glioma cells. Heightened CD44 expression has been shown to play a key role in glioma recurrence, as it is associated with invasion and proliferation of tumour cells by initiating cellular signalling pathways that give rise to the highly invasive glioma stem cell phenotype [24]. High expression of CD44 is also associated with poor prognosis and early tumour recurrence in glioma patients, compared to those with lower CD44 expression [25]. This also supports the significant increase seen in glioma cell density when in co-culture. It has been previously shown that astrocytes actively facilitate glioma proliferation and invasion by activating CD44 signalling pathways, which induces tumour cell transformation to the highly invasive and proliferative glioma stem cell phenotype [26]. Further studies using proliferation assays such as BrdU or Ki67 staining would likely shed more light onto this seemingly increased proliferative state of glioma cells in co-culture with astrocytes.

**Fig. 7:**
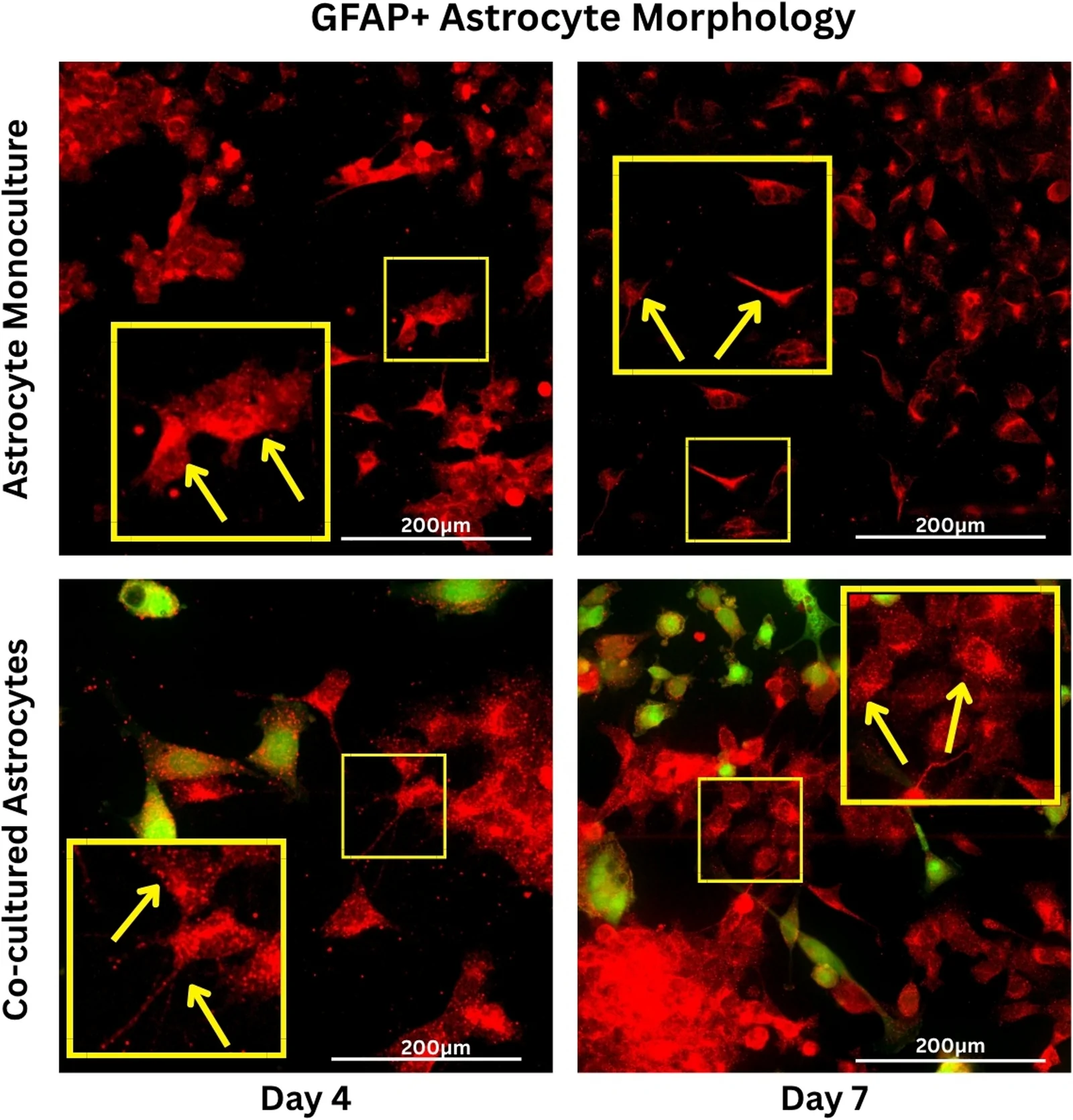
Qualitative analysis of astrocyte morphology in GFAP + astrocytes in monoculture and co-culture samples. Yellow arrows highlight astrocytes of interest in both culture conditions. Astrocytes with longer and a higher number of processes are indicative of A1 (inflammatory) phenotype and seem to be more prevalent in astrocytes grown in co-culture. Rounder cell bodies and fewer processes are indicative of A2 (neuroprotective) phenotype and are more prevalent in astrocytes grown in monoculture

Additionally, this model showed that the relative intensity of GFAP in astrocytes increased significantly when co-cultured with glioma cells, whereas astrocyte monocultures did not see a similar increase over time. GFAP is a marker for reactive astrocytes whereby astrocytes transform in response to cellular injury, stress or disease [27]. Other studies suggest that the presence of reactive astrocytes is involved in regulation of glioma progression through many mechanisms such as mitochondrial transfer, but primarily through direct cell-to-cell interaction with tumour cells which enhances their survival, proliferation and invasion into healthy tissue [28]. Qualitative analysis of astrocyte morphology suggests that astrocytes in monoculture present primarily in the A2 (neuroprotective) phenotype, whereas contact with glioma cells in co-culture seem to shift the morphology towards an A1 (inflammatory) phenotype (Fig. 7) [29, 30]. This is consistent with the upregulation of GFAP over time seen only in astrocytes which were co-cultured with glioma cells. Another intriguing qualitative finding from this work is that astrocytes appear to selectively cluster away from glioma cells during glioma invasion (Figure S8). Further work could illustrate if this is due to selective apoptosis of astrocytes in contact with glioma or due to protective migration away from the invading glioma.

### Advantages of microfluidic platform

A notable advantage of the platform developed in this work is that it allows for precise control over directional spheroid growth by forcing growth into the directional microfluidic channels. This presents a significant advantage over co-cultures grown inside of circular multi-well plates. Recent work in this area has performed spheroid co-cultures in multi-well plates, but have illustrated how spheroid growth is multi-directional, meaning outgrowth measurements cannot be accurately quantified by measurements taken from a set boundary in a single direction [22]. As a comparison, the same co-culture used here (i.e. glioma spheroid and astrocyte monolayer) was compared to growing in a stand-alone 96-well plate and imaged. The spheroids exhibited outgrowth; however, it was sporadic and inconsistent around the periphery, highlighting the use-case for microchannels guiding outgrowth to aid in accurate measurements (Figure S6). Additionally, although gas permeable PDMS was used in this platform, a gas impermeable material such as poly (methyl methacrylate) could be used to exert further control over the culture environment by creating more or less hypoxic regions which could lead to increased glioma outgrowth.

### Critical evaluations and future directions

#### Cell type

As this work was primarily for model optimisation, rat cells which are less physiologically relevant but more easily grown, served to validate the functionality and biological relevance of the microfluidic platform. The translation of this platform for use with human cells is already underway as there is a clear benefit for pushing this model towards clinical relevance through incorporating patient derived cells in the future. Furthermore, these experiments only included astrocytes as the non-tumour cells, as this work served to provide evidence to support the feasibility of the developed microfluidic platform. In the future, the inclusion of other neural cell types such as neurons and microglia will further highlight additional cellular interactions with tumour cells [31–33]. In addition to different cell types, using this platform to test cells from different tumour locations (i.e. within necrotic core versus tumour periphery), could shed light on cellular mechanisms leading to the extreme heterogeneity seen in glioblastoma tumours.

#### Two-dimensionality

An additional limitation is that although this model included three-dimensional elements such as spheroids, the outgrowth was confined to a two-dimensional space along the bottom of a microfluidic channel. This could have significantly altered the results in comparison to a fully three-dimensional model as glioblastoma is known to spread aggressively in three dimensions. A simpler two-dimensional astrocyte culture and channel configuration was used here to facilitate the optimisation of the in vitro model system, however in the future, the incorporation of a hydrogel into the model could lead to important improvements in recapitulating the neural microenvironment [34].

#### Flow

Finally, the current platform did not incorporate flow as a primary component, however it is clear that fluid flow has profound effects on both perceived cell stresses and nutrient availability in microfluidic culture systems [35]. Therefore, future implementations of this system will be trialled with incorporating perfusion either through peristaltic pumping [36] or rocking [37].

## Conclusion

This work has outlined the developmental stages and proof-of-concept for an on-chip model of glioma recurrence. Using microfluidic channels to guide glioma outgrowth from a spheroid, significant differences were seen in glioma growth when in co-culture versus in monoculture. Additionally, significant differences were seen in the presence of pro-inflammatory and proliferative markers in astrocytes and glioma cells respectively. The initial data presented in this study demonstrated that the developed microfluidic platform can be used as a practical and viable method for modelling glioma invasion into healthy tissue and provided further evidence toward visualizing cellular crosstalk and its important role in cancer cell migration and metastasis. The ability to model these changes in an in vitro system is promising for the further development of models to shed light on the mechanisms underpinning glioblastoma recurrence.

## Supplementary Information

Below is the link to the electronic supplementary material.

ESM 1 (DOCX 6.50 MB)

## Data Availability

All analysis methods are provided in the manuscript and supplementary information. Raw data including images and analyzed numberical data is available upon request.

## References

[1] Louis DN, Perry A, Reifenberger G, Von Deimling A, Figarella-Branger D, Cavenee WK, et al. The 2016 world health organization classification of tumors of the central nervous system: a summary. Acta Neuropathol. 2016;131:803–20. 10.1007/s00401-016-1545-1 27157931

[2] Woodworth, Davis C, Whitney B, Pope LM, Liau HJ, Kim A, Lai PL, Nghiemphu, Timothy F, Cloughesy, Benjamin M. Ellingson. Nonlinear distortion correction of diffusion MR images improves quantitative DTI measurements in glioblastoma. J Neuro-oncol. 2014;116:551–558. 10.1007/s11060-013-1320-2 PMC3918907 24318915

[3] Lenting, Krissie R, Verhaak MT, Laan P, Wesseling, William Leenders. Glioma: experimental models and reality. Acta Neuropathol. 2017;133:263–82. 10.1007/s00401-017-1671-4 PMC5250671 28074274

[4] National Institute for Health and Care Excellence (NICE). Brain tumours (primary) and brain metastases in over 16s: NICE guidelines. NG99; 2019. Available at: https://www.nice.org.uk/guidance/ng99/resources 31393680

[5] - Mahvi DA, Liu R, Grinstaff MW, Colson YL, Chandrajit P. Raut. Local cancer recurrence: the realities, challenges, and opportunities for new therapies. Cancer J Clin. 2018;68(6):488–505. 10.3322/caac.21498 PMC6239861 30328620

[6] Palmer G, Martling A, Cedermark B, Holm T. A population-based study on the management and outcome in patients with locally recurrent rectal cancer. Ann Surg Oncol. 2007;14:447–54. 10.1245/s10434-006-9256-9 17139457

[7] - Warren JL, Robin Yabroff K. Challenges and opportunities in measuring cancer recurrence in the united States. J Natl Cancer Inst. 2015;107:8. 10.1093/jnci/djv134 PMC4580558 25971299

[8] Atat OE, Farzaneh Z, Pourhamzeh M, Taki F, Abi-Habib R, Vosough M, El-Sibai M. 3D modeling in cancer studies. Hum Cell. 2022;35(1):23–36. 10.1007/s13577-021-00642-9 34761350

[9] Atat O, El Z, Farzaneh M, Pourhamzeh F, Taki, Paolillo, Mayra S, Comincini, Schinelli S. Paolillo, Mayra, Sergio, Comincini, Sergio Schinelli. In vitro glioblastoma models: a journey into the third dimension. Cancers. 2021;13(10):2449. 10.3390/cancers13102449 PMC8157833 34070023

[10] Edmondson, Rasheena JJ, Broglie, Audrey F Adcock, Liju Yang. Three-dimensional cell culture systems and their applications in drug discovery and cell-based biosensors. Assay and Drug Dev Technol. 2014;12(4):207–218. 10.1089/adt.2014.573 PMC4026212 24831787

[11] - Suva ML, Itay Tirosh. The glioma stem cell model in the era of single-cell genomics. Cancer Cell. 2020;37(5):630–6. 10.1016/j.ccell.2020.04.001 32396858

[12] Diao W, Tong X, Yang C, Zhang F, Bao C, Chen H, et al. Behaviors of glioblastoma cells in *in vitro* microenvironments. Sci Rep. 2019;9(1):85. 10.1038/s41598-018-36347-7 PMC6331579 30643153

[13] - DePalma, Thomas J, Sivakumar H, Aleksander Skardal. Strategies for developing complex multi-component in vitro tumor models: highlights in glioblastoma. Adv Drug Deliv Rev. 2022;180:114067. 10.1016/j.addr.2021.114067 PMC10560581 34822927

[14] - Joseph JV, Mathilde S, Blaavand T, Daubon FAE, Kruyt, Martin K. Thomsen. Three-dimensional culture models to study glioblastoma—Current trends and future perspectives. Curr Opin Pharmacol. 2021;61:91–7. 10.1016/j.coph.2021.08.019 34656940

[15] - Sung K, Eun, Beebe DJ. Microfluidic 3D models of cancer. Adv Drug Deliv Rev. 2014;79:68–78. 10.1016/j.addr.2014.07.002 PMC4258433 25017040

[16] Chauhdari T, Su J, Lv J, Ding Y. Ecm-free lung tumoroids generated by an air-stretchable microfluidic chip. In vitro models. 2025;4:1–3. 10.1007/s44164-025-00093-8 PMC12957707 41788237

[17] - Mansoury, Morva M, Hamed R, Karmustaji FA, Hannan, Stephen T. Safrany. The edge effect: A global problem. The trouble with culturing cells in 96-well plates. Biochem Biophys Rep. 2021;26:100987. 10.1016/j.bbrep.2021.100987 PMC8024881 33855228

[18] Bach C, Glasow A, Baran-Schmidt R, Oppermann H, Bach C, Meixensberger J, et al. Rapid and reproducible generation of glioblastoma spheroids for high-throughput drug screening. Front Bioeng Biotechnol. 2024;12:p1471012. 10.3389/fbioe.2024.1471012 PMC11688379 39744592

[19] Mirab Fereshtehsadat YJ, Kang Majd S. Preparation and characterization of size-controlled glioma spheroids using agarose hydrogel microwells. PLoS One. 2019;14(1):e0211078. 10.1371/journal.pone.0211078 PMC6345430 30677075

[20] - Mourad PD, Farrell L, Stamps LD, Santiago P, Fillmore HL, Broaddus WC, Silbergeld DL. Quantitative assessment of glioblastoma invasion in vivo. Cancer Lett. 2003;192(1):97–107. 10.1016/s0304-3835(02)00637-7 12637158

[21] Oraiopoulou ME, Tampakaki M, Tzamali E, Tamiolakis T, Makatounakis V, Vakis AF, et al. A 3D tumor spheroid model for the T98G glioblastoma cell line phenotypic characterization. Tissue Cell. 2019;59:39–43. 10.1016/j.tice.2019.05.007 31383287

[22] Förster N, Isosaari L, Kulta O, Junnila O, Vuolanto V, Pollari M, et al. Functional 3D human neuron–glioblastoma model reveals cellular interactions enabling drug safety assessments. FASEB J. 2025;39(8):e70567. 10.1096/fj.202500291RR PMC12023715 40277152

[23] Gagliano N, Costa F, Cossetti C, Pettinari L, Bassi R, Chiriva-Internati M, Cobos E, Magda Gioia, Stefano Pluchino. Glioma-astrocyte interaction modifies the astrocyte phenotype in a co-culture experimental model. Oncol Rep. 2009;22(6):1349–1356. 10.3892/or_00000574 19885586

[24] Inoue A, Ohnishi T, Nishikawa M, et al. A narrative review on CD44’s role in glioblastoma invasion, proliferation, and tumor recurrence. Cancers (Basel). 2023;15(19):4898. 10.3390/cancers15194898. PMC10572085 37835592

[25] Nishikawa M, Inoue A, Ohnishi T, Kohno S, Ohue S, Matsumoto S, Suehiro S et al. Significance of glioma stem-like cells in the tumor periphery that express high levels of CD44 in tumor invasion, early progression, and poor prognosis in glioblastoma. Stem Cells Int. 2018;201(1):5387041. 10.1155/2018/5387041 PMC6126065 30210550

[26] Du Z, Wang Y, Liang J, et al. Association of glioma CD44 expression with glial dynamics in the tumour microenvironment and patient prognosis. Comput Struct Biotechnol J. 2022;20:5203–17. 10.1016/j.csbj.2022.09.003. PMC9508470 36187921

[27] Pekny Milos, Wilhelmsson Ulrika, Pekna Marcela. The dual role of astrocyte activation and reactive gliosis. Neurosci Lett. 2014;565:30–8. 10.1016/j.neulet.2013.12.071 24406153

[28] Wu J, Li R, Wang J, et al. Reactive astrocytes in glioma: emerging opportunities and challenges. Int J Mol Sci. 2025;26(7):2907. 10.3390/ijms26072907. PMC11989224 40243478

[29] Fan YY, Huo J. A1/A2 astrocytes in central nervous system injuries and diseases: angels or devils? Neurochem Int. 2021;148:105080. 10.1016/j.neuint.2021.105080 34048845

[30] Zou LH, Shi YJ, He H, Jiang SM, Huo FF, Wang XM, et al. Effects of FGF2/FGFR1 pathway on expression of A1 astrocytes after infrasound exposure. Front Neurosci. 2019;13:429. 10.3389/fnins.2019.00429 PMC6509904 31130839

[31] Goshi N, Morgan RK, Lein PJ, et al. A primary neural cell culture model to study neuron, astrocyte, and microglia interactions in neuroinflammation. J Neuroinflammation. 2020;17:155. 10.1186/s12974-020-01819-z. PMC7216677 32393376

[32] Song H, Bucher S, Rosenberg K, Tsui M, Burhan D, Hoffman D, et al. Single-cell analysis of hepatoblastoma identifies tumor signatures that predict chemotherapy susceptibility using patient-specific tumor spheroids. Nat Commun. 2022;13(1):4878. 10.1038/s41467-022-32473-z PMC9411569 36008377

[33] - Manini I, Caponnetto F, Bartolini A, Ius T, Mariuzzi L, Loreto CD, Beltrami AP, Daniela Cesselli. Role of microenvironment in glioma invasion: what we learned from in vitro models. Int J Mol Sci. 2018;19(1):147. 10.3390/ijms19010147. PMC5796096 29300332

[34] Matthiesen I, Jury M, Rasti Boroojeni F, Ludwig SL, Holzreuter M, Buchmann S, et al. Astrocyte 3D culture and bioprinting using peptide functionalized hyaluronan hydrogels. Sci Technol Adv Mater. 2023. 10.1080/14686996.2023.2165871. PMC9888471 36733710

[35] Sato K, Sato M, Yokoyama M, Hirai M, Furuta A. Influence of culture conditions on cell proliferation in a microfluidic channel. Anal Sci. 2019;35(1):49–56. 10.2116/analsci.18SDP04 30473567

[36] - Aljayyousi H, Sahloul S, Orozaliev A, Baban N, Van AD, Al Nuairi A, John P, Zam A, Percipalle P, Song YA. A modular and reconfigurable microfluidic device for culturing spheroids under continuous perfusion. APL Bioeng. 2025;9(3):036111. PMID: 40821387; PMCID: PMC12352930. 10.1063/5.0262536 PMC12352930 40821387

[37] Limjanthong N, Sugiura S, Oda T, Takusari F, Fujiwara Y, Miyazaki T, et al. Monitoring and optimization of the microenvironment in a gravity-driven microfluidic system placed on a slow-tilting table. J Biosci Bioeng. 2025;139(4):311–8. 10.1016/j.jbiosc.2024.12.014 39843315

