# Supplementary material for: Development and characterization of an on-chip glioma outgrowth model
Source: In Vitro Model. 2025 Dec 8;5(4):299–311. doi: 10.1007/s44164-025-00100-y (PMC13768485; doi:10.1007/s44164-025-00100-y)
Supplement: Supplementary file 1 [file 44164_2025_100_MOESM1_ESM.docx]

**Supplementary Information for**

**Development and characterization of an on-chip glioma outgrowth model**

Joshua P. A. Daoud^1^, Alexandra E. Collisson^1^, Daniya Asaid^2^, Ester Clarisse do Couto Lopes^1^, Dimitrios Paraskevopoulos^3^, Christopher A. R. Chapman^1*^

1. School of Engineering and Materials Science, Queen Mary University of London, U.K.
2. Blizard Institute, Queen Mary University of London, London, United Kingdom
3. Department of Neurosurgery, The Royal London Hospital, Barts Health NHS Trust, London, United Kingdom

* Corresponding author: Christopher A. R. Chapman – christopher.chapman@qmul.ac.uk

**Materials**

Cell Lines:

Rat glioma cells – 9L/3cmv-GFP cell line

Rat glioma cells – 9L/lacZ cell line

Rat astrocytes – CTX TNA2 cell line – UK Health Security Agency

Cell culture:

Dulbecco's Modified Eagle Medium (DMEM) – ThermoFisher Scientific

Dulbecco’s Phosphate buffered saline (D-PBS) - ThermoFisher scientific

Corning 96-well spheroid microplate – Sigma-Aldrich

Fetal Bovine Serum – ThermoFisher Scientific

Poly-D-Lysine - ThermoFisher Scientific

Laminin - ThermoFisher Scientific

Staining:

Paraformaldehyde (PFA) 4% - Sigma-Aldrich

Triton^™^ X-100 – Sigma-Aldrich

Bovine Serum Albumin (BSA) – ThermoFisher Scientific

DAPI 10mg – ThermoFisher Scientific

Texas-Red Phalloidin – ThermoFisher Scientific

Normal Goat Serum – ThermoFisher Scientific

Live-or-Dye NucFix™ Red Staining Kit - Biotium

Antibodies:

CD44 Recombinant Rabbit Monoclonal Antibody - Invitrogen

GFAP Mouse Monoclonal Antibody (GA5) – Invitrogen

Cy^™^5 AffiniPure Goat-Anti-Rabbit IgG – Jackson ImmunoResearch

Cy^™^3 AffiniPure Goat-Anti-Mouse IgG - Jackson ImmunoResearch

Additional Equipment:

Nikon CSU-W1 SoRa Spinning Disc Confocal microscope

Nikon Elements Viewer software

Profilometer: Profilm3D^®^

ImageJ (Fiji) Computer Program

SYLGARD 184 Silicone Elastomer Kit

KORASILON-paste – Sigma-Aldrich

**Experimental Optimisations**

*Optimisation 1:* Due to the porosity of the PDMS material, this initially led to excessive media evaporation and air bubble formation, leading to loss of cell viability. To remedy this, microfluidic chips were pre-incubated with DMEM media for 24 hours before cell seeding.

*Optimisation 2:* Media changes were carried out once every 48 hours for the first two changes, then increased to once every 24 hours. This was to allow adequate time for cells to adhere and grow so that they did not detach during future media changes.

**Supplementary Figures**

**Figure S1:** Image displaying the set-up of microfluidic chips inside of a glass petri dish including the PDMS rings added to channel surface. Small reservoirs of sterile D-PBS were also kept inside of the petri dish throughout the duration of the experiment. These steps prevented excessive media evaporation, enabling longer experimental running times.

**Figure S2** Experimental timeline for co-culture and control experiments inside of the microfluidic platform, including the frequency of culture media replenishment.

**Figure S3** Images showing how the spheroid outgrowth experimental data was calculated from fluorescent imaging data which was analysed inside of ImageJ software.

Spheroid outgrowth measurements were taken using ImageJ to analyse fluorescent images. As the outgrowth was purely obtained from spheroids, only GFP channels were used to identify the glioma cells, which had grown out into the microfluidic channel. Firstly, the entrance to the microfluidic channel was identified, and measurements were taken from the channel entrance to any visible cells which have grown to the furthest observable regions of outgrowth on the image, as shown in the top image. For each sample, measurements were taken of the 20 furthest visible points of outgrowth on each side of the channel (n = 40), which were averaged to produce a mean outgrowth value.

**Figure S4** Images showing how the cell density experimental data was quantified using fluorescent imaging inside of ImageJ software.

Cell density data was calculated from fluorescent images showing the nuclear DNA, stained with DAPI nuclear stain, for all culture types. Within ImageJ, a set size region of the image was selected (0.2mm^2^), and the cell population was counted in the designated area of the image using ImageJ functions. This was repeated across several different regions of a given image to provide an average cell count for the culture type. Once the average cell counts per 0.2mm^2^ area were calculated, the image area was scaled up by a factor of 5 to produce cell density values for the number of cells/mm^²^.

**Figure S5** Images showing how the antibody staining experimental data was quantified using fluorescent imaging inside of ImageJ software.

Relative intensities of antibody levels for CD44 and GFAP were quantified using the “ROI” and “measure” functions in ImageJ. For control samples, a region of interest (ROI) was applied to a single cell (top right image), and the same ROI was applied to the colour channel, which corresponded with staining with the antibody of interest (top left image). The “measure” tool in ImageJ was applied to the ROI to obtain a relative value of colour intensity. The ROI measurements were repeated 10 times for each image and averaged to provide a mean intensity value. In co-culture samples, the same method was applied, except separate ROIs were applied to glioma cells and astrocyte cells to assess which cell type may have been responsible for an increased relative intensity of the markers of interest.

**Figure S6:** GFP signal (glioma cells – 9L/3cmv-GFP) from supplementary co-culture experiments which were carried out in a 6-well plate. The images illustrate how spheroid outgrowth is measured but due to the multi-directional growth, outgrowth cannot be easily measured from a set starting point.

**Figure S7:** Spheroid placement analysis showing how spheroids were inserted into the well of the microfluidic chip. A circle showing the centre of the well has been placed on the image to determine the accuracy of spheroid placement across three example images from different samples.

**Figure S8:** Clustering of astrocytes around ingrowth of glioma cells in the microfluidic channel.
